# High correlation of VAS pain scores after 2 and 6 weeks of treatment with VAS pain scores at 12 weeks in randomised controlled trials in rheumatoid arthritis and osteoarthritis: meta-analysis and implications

**DOI:** 10.1186/s13075-016-0972-7

**Published:** 2016-03-31

**Authors:** Andreas Karabis, Stavros Nikolakopoulos, Shaloo Pandhi, Katerina Papadimitropoulou, Richard Nixon, Ricardo L. Chaves, R. Andrew Moore

**Affiliations:** Real World Strategy & Analytics, Mapi Group, Houten, The Netherlands; Novartis Pharma AG, Basel, Switzerland; Pain Research, Nuffield Division of Anaesthetics, University of Oxford, The Churchill, Oxford, OX3 7LE UK

**Keywords:** Rheumatoid arthritis, Osteoarthritis, NSAID, VAS pain

## Abstract

**Background:**

Researchers in clinical trials in rheumatoid arthritis (RA) and osteoarthritis (OA) often measure pain levels with a visual analogue scale (VAS). Of interest to clinical practice and future clinical trial design are associations of change from baseline (CFB) between time points with predictive ability of earlier response for long-term treatment benefit. We assessed the association and predictive ability of CFB in VAS pain between 2, 6 and 12 weeks in randomised controlled trials (RCTs) of non-steroidal anti-inflammatory drugs (NSAIDs).

**Methods:**

Aggregated VAS pain data at baseline and CFB at 2, 6 and 12 weeks were collected from a systematic literature review of 176 RCTs in OA and RA. The predictive ability of earlier assessments for longer-term pain reduction was estimated using correlation and regression analyses. Analysis was performed using the R software package for statistical programming, version 3.1.1.

**Results:**

Appropriate data were available from 50 RCTs (22,854 patients). Correlations between time points were high (weighted correlation coefficients between 2 and 6 weeks, 0.84; between 2 and 12 weeks, 0.79; and between 6 and 12 weeks, 0.96). CFB at 6 weeks was highly predictive and close to CFB at 12 weeks (regression coefficient 0.9, 95 % confidence interval 0.9–1.0). CFB at 2 weeks was significantly associated with CFB at 12 (0.8, 0.7–0.8) and 6 weeks (0.9, 0.8–1.0).

**Conclusions:**

The results showed that early analgesic response measured by VAS for pain beyond 2 weeks of treatment with a particular NSAID is likely to be predictive of response at 12 weeks. Failure to achieve desired pain relief in OA and RA after 2 weeks should trigger reassessment of dosage and/or analgesic.

**Electronic supplementary material:**

The online version of this article (doi:10.1186/s13075-016-0972-7) contains supplementary material, which is available to authorized users.

## Background

Osteoarthritis (OA) and rheumatoid arthritis (RA) are the most common arthritic conditions in adults [1]. They lead to joint degeneration and chronic pain [2, 3]. Their prevalence is increasing with an aging population [4], and pain management is a global public health priority [5, 6]. Worldwide, OA is the 11th most common condition causing long-term disability [7].

Non-steroidal anti-inflammatory drugs (NSAIDs), both traditional and cyclooxygenase 2 inhibitors (COXIBs), are commonly prescribed to relieve pain and inflammation [2, 3] and are the cornerstones for treating pain in arthritis [8, 9]. Knowledge of their effectiveness derives from randomised trials and meta-analyses of randomised trials. In these trials, pain is typically measured using a visual analogue scale (VAS) or a categorical scale. While clearly not the only outcome of interest, pain is an important efficacy outcome in OA and RA trials and perhaps the one most important to patients [10–13].

Recent clinical trials have follow-up periods of 12 weeks, which has become a regulatory requirement for registration of symptom-modifying drugs in OA [14]. Change from baseline (CFB) in pain levels is often assessed at multiple time points to evaluate efficacy, though analyses at the level of the individual patient have also become available in some cases [15]. In analyses of efficacy, researchers have tended to express their results as those at 12 weeks, and they often comment briefly, if at all, on the dynamics of changes over time. Particularly missing from the literature is evidence relating later, and presumably ongoing, benefit to early benefit. Limited previous work on OA has indicated that early response is predictive of later response [16], and there are supportive findings in fibromyalgia and acute pain [17, 18].

While many clinicians may recognise the link between early and late pain response and non-response in their everyday practice, this tends not to be reflected in guidance. For example, if early non-response predicted that there would be no later response, guidance might well suggest early switching of therapy in the face of non-response after 2, 4 or 6 weeks. This does not happen, and, as a consequence, perhaps pain is frequently poorly treated: over half of patients still had moderate or severe pain despite being on treatment [19].

This study builds on a large, recent systematic review and network meta-analysis of traditional NSAIDs and COXIBs in patients with OA or RA in which researchers examined relative benefits and risks [20]. The data collected in that systematic review were derived from 146,524 patients in 176 studies and offered a unique opportunity for additional analyses.

In the present study, we therefore set out to assess the association and predictive ability of CFB in VAS pain scores between the time points of 2, 6 and 12 weeks in randomised controlled trials (RCTs) including traditional NSAIDs, COXIBs and placebo. The predictive ability of earlier pain measurements for long-term treatment benefit is of interest to clinical practice and future clinical trial design, and would add substantially to the currently available literature in arthritis.

## Methods

The evidence base for this analysis was obtained from a recently published systematic literature review that included 176 RCTs with a total of 146,524 patients with OA and RA [20]. This review was conducted in June 2013 using MEDLINE, Embase and the Cochrane Library to identify RCTs comparing diclofenac, ibuprofen, naproxen, celecoxib or etoricoxib with each other or with placebo. Efficacy data were collected, including pain relief measured with the VAS or the Western Ontario McMaster Universities Arthritis Index VAS at three time points: 2, 6 and 12 weeks of treatment.

To assess the association between mean CFB in VAS pain scores, a linear regression model was fitted for all the possible pairs of subsequent measurements, with the later time point as an outcome and the earlier time point as the predictor. Thus, three models were fitted to the respective available data: model 1, predicting mean CFB at 6 weeks based on mean CFB at 2 weeks; model 2, predicting mean CFB at 12 weeks based on mean CFB at 2 weeks; and model 3, predicting mean CFB at 12 weeks based on mean CFB at 6 weeks.

Each treatment arm was treated as a data point, while random intercept models were fitted to account for clustering within studies.

As the number of patients analysed in each of the included studies varied considerably, the weight of each data point should be taken into consideration proportionally. Thus, two weighting options were tested for these models: (1) weighting by the sample size used at the later measurement and (2) weighting by the precision of the later measurement, implying the outcome variable of each model. The Akaike information criterion (AIC) was used for selecting the model option that best fit the data. Models with a smaller AIC are better supported by the data. R software for statistical programming [21], version 3.1.1, was used for the analysis.

Correlation coefficients (Pearson’s *r*) are presented with the weighting method used. This is a measure of the linear correlation between two variables, *x* and *y*, giving a value between +1 and −1 inclusive, where 1 is total positive correlation, 0 is no correlation and −1 is total negative correlation.

## Results

The evidence base used for our analysis consisted of 50 RCTs (from the 176 identified RCTs in the original literature review [20]) in which CFB was reported for at least two of the time points (2, 6 or 12 weeks) of interest. All these RCTs were included in the regression modelling, as they reported both the sample size and the standard error of the estimates for at least one of the time points of interest and thus weighted regression models could be estimated. The individual study results used for our analyses are presented in Additional file 1. Details on study design and patient characteristics of all included studies are provided in a previously published article [20] and in Additional files 2 and 3, respectively.

Overall, 33 RCTs (76 arms, 14,919 patients) reported VAS pain scores at 2 and 6 weeks (model 1), 21 RCTs (54 arms, 12,618 patients) at 2 and 12 weeks (model 2) and 26 RCTs (63 arms, 14,643 patients) at 6 and 12 weeks (model 3). Descriptive statistics on CFB in VAS pain scores at weeks 2, 6 and 12 are shown in Table 1. These data indicate that the mean and median CFB in VAS pain scores had only limited variation over time.

**Table 1:** Descriptive statistics for change from baseline in visual analogue scale pain

VAS pain	Mean	Median	Minimum	Maximum
CFB at 2 weeks	−21.0	−21.8	−35.5	−0.3
CFB at 6 weeks	−23.5	−24.5	−46.9	−1.0
CFB at 12 weeks	−21.0	−21.9	−42.9	1.8

Sample size weighted correlation coefficients between the three time points are presented in Table 2. Pearson’s *r* values are 0.84 between 2 and 6 weeks, 0.79 between 2 and 12 weeks, and 0.96 between 6 and 12 weeks. This indicates a very strong positive association between outcomes at the evaluated time points, and that for most patients early and later response or non-response will be much the same, with few experiencing a different late response compared with the early response. Clinical effect (decrease in VAS pain score) observed at the earlier time points (i.e., 2 or 6 weeks) of treatment is associated with the effect (decrease in VAS pain score) at the later time points (i.e., 6 or 12 weeks). Thus, clinical effect (decrease in VAS pain score) observed at the earlier time points (i.e., 2 or 6 weeks) of treatment is predictive of the effect at the later time points (i.e., 6 or 12 weeks).

**Table 2:** Sample size weighted Pearson correlation coefficients (*r* values) for change from baseline in visual analogue scale pain

Time point	2 weeks	6 weeks	12 weeks
2 weeks	1		
6 weeks	0.84	1	
12 weeks	0.79	0.96	1

The (average) intercept and slope, together with the 95 % confidence interval (CI) and AIC for each model, are reported in Table 3. For models 1 and 3, the AIC was lower when weighted by sample size, and we focus on these results below. For model 2, the AIC values were very close and thus the sample size weighted model was chosen for consistency. The observed versus fitted values and the corresponding residuals for each model are presented in Additional file 4.

**Table 3:** Weighted regression models for change from baseline in visual analogue scale pain

Model	Predictor	Outcome	N-weighted			SE-weighted		
			Slope (95 % CI)	Intercept (95 % CI)	AIC	Slope (95 % CI)	Intercept (95 % CI)	AIC
1	2 weeks	6 weeks	0.9 (0.8–1.0)	−4.6 (−6.9, −2.4)	394	0.9 (0.8–1.0)	−4.0 (−6.8, −1.1)	450
2	2 weeks	12 weeks	0.8 (0.7–0.8)	−8.3 (−10.4, −6.2)	256	0.7 (0.7–0.8)	−8.5 (−10.4, −6.5)	256
3	6 weeks	12 weeks	0.9 (0.9–1.0)	−1.5 (−3.1, 0.2)	253	0.9 (0.9–1.0)	−1.2 (−2.8, 0.3)	261

*AIC* Akaike information criterion, *CI* confidence interval, *N* sample size, *SE* standard error

### Predicting average CFB in VAS pain score at 6 weeks

**Fig. 1:**
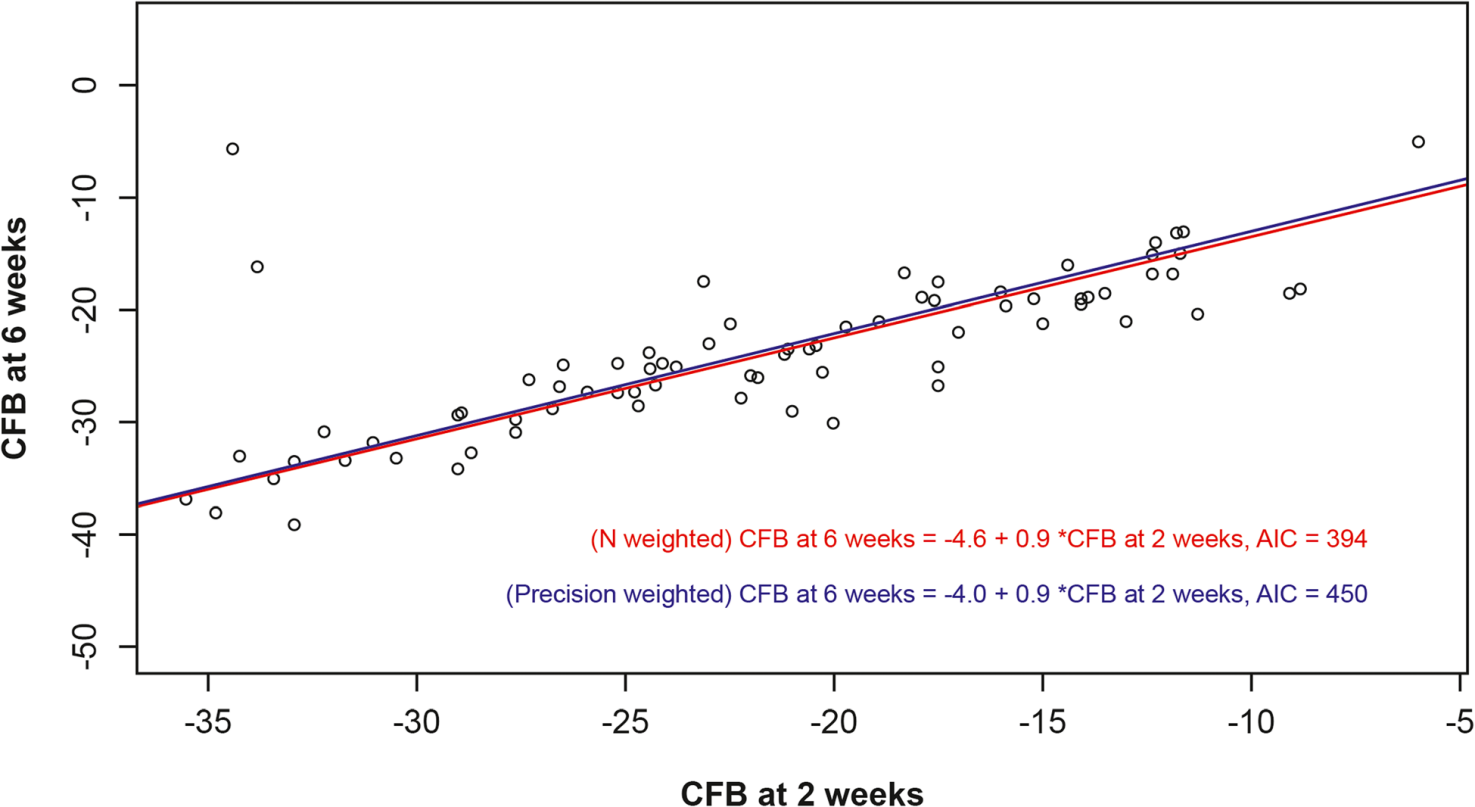
CFB in VAS pain 2-week data versus CFB in VAS pain 6-week data. A scatterplot of observed CFB data from RCT arms is displayed along with predicted regression lines, N-weighted (*red line*) and precision-weighted (*blue line*). *AIC* Akaike information criterion; *CFB* change from baseline, *RCT* randomised controlled trial, *VAS* visual analogue scale

CFB in VAS pain score at 2 weeks was significantly associated with CFB in VAS pain score at 6 weeks (regression coefficient 0.9, 95 % CI 0.8–1.0); intercept −4.6, 95 % CI −6.9, −2.4). A scatterplot of observed data per arm at both time points, along with predicted regression lines N-weighted and precision-weighted, is presented in Fig. 1.

### Predicting average CFB in VAS pain score at 12 weeks

**Fig. 2:**
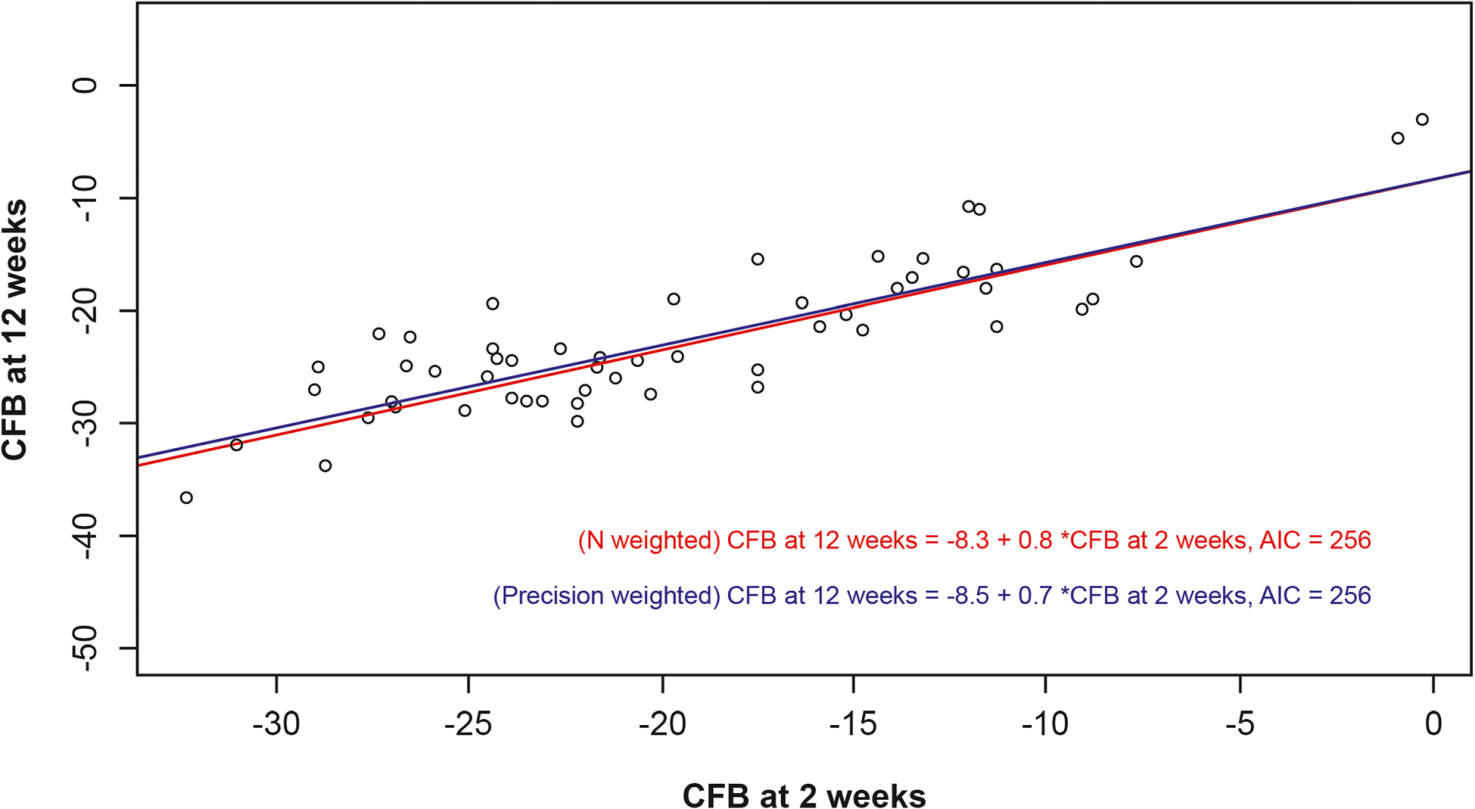
CFB in VAS pain 2-week data versus CFB in VAS pain 12-week data. A scatterplot of observed CFB data from RCT arms is displayed along with predicted regression lines, N-weighted (*red line*) and precision-weighted (*blue line*). *AIC* Akaike information criterion; *CFB* change from baseline, *RCT* randomised controlled trial, *VAS* visual analogue scale

**Fig. 3:**
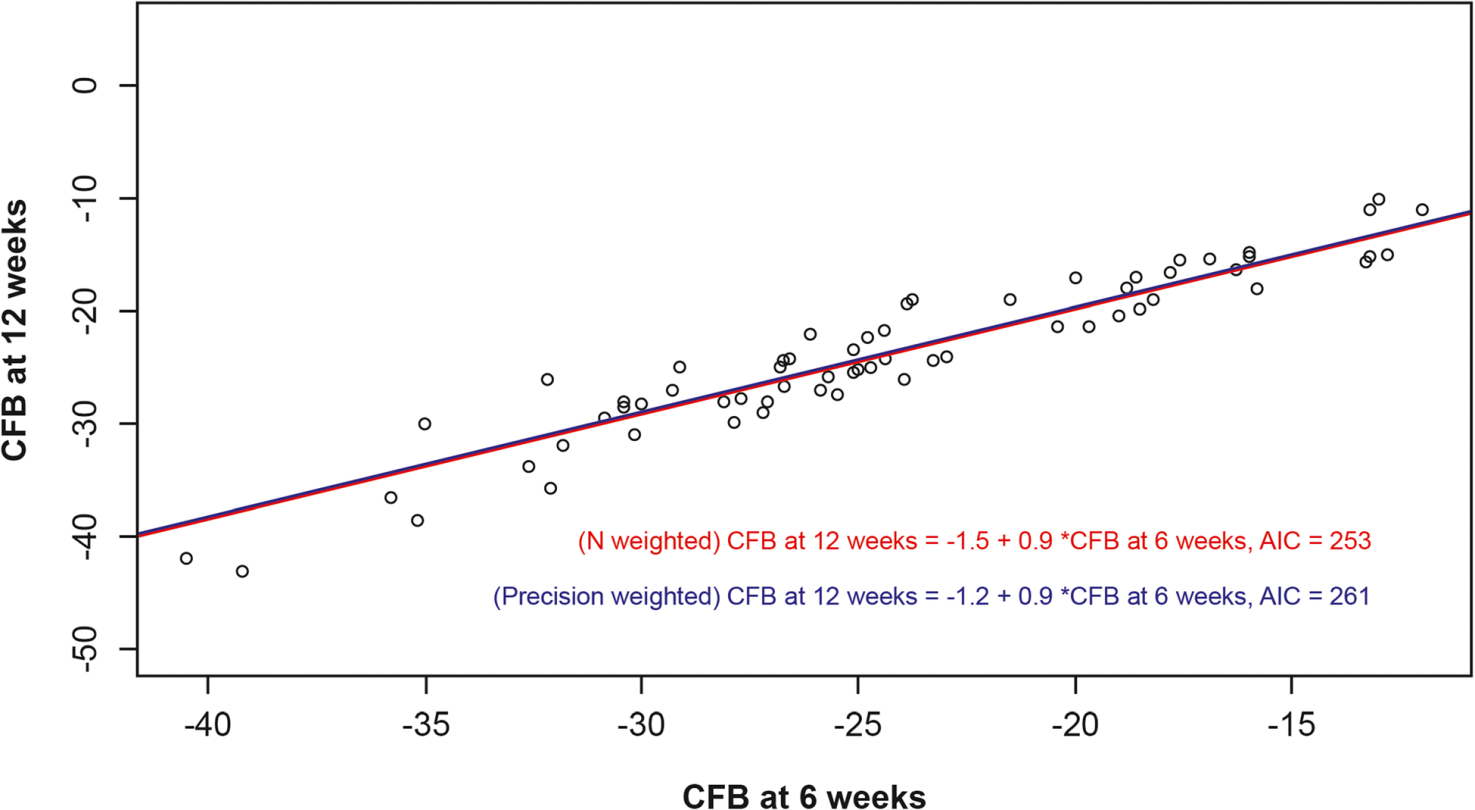
CFB in VAS pain 6-week data versus CFB in VAS pain 12-week data. A scatterplot of observed CFB data from RCT arms is displayed along with predicted regression lines, N-weighted (*red line*) and precision-weighted (*blue line*). *AIC* Akaike information criterion; *CFB* change from baseline, *RCT* randomised controlled trial, *VAS* visual analogue scale

CFB in VAS pain score at 2 weeks was significantly associated with CFB in VAS pain score at 12 weeks (regression coefficient 0.8, 95 % CI 0.7–0.8; intercept −8.3, 95 % CI −10.4, −6.2). Similarly, CFB in VAS pain score at 6 weeks was found to be highly predictive and very close to CFB in VAS pain score at 12 weeks (regression coefficient 0.9, 95 % CI 0.9–1.0; intercept −1.5, 95 % CI −3.1, 0.2). Scatterplots of observed CFB in VAS pain data per arm at 2 and 12 weeks and 6 and 12 weeks, along with the predicted regression lines, are presented in Figs. 2 and 3, respectively.

## Discussion

In this study, we assessed the association and predictive ability of CFB in VAS pain score between the time points of 2, 6 and 12 weeks in RCTs of OA and RA. The analysis was based on data derived from a systematic literature review of published RCTs comparing diclofenac, ibuprofen, naproxen, celecoxib or etoricoxib with each other or with placebo [20]. That review provided a unique opportunity to explore the relationship between early and late pain responses in RCTs.

The results suggest that average CFB in VAS pain scores at all time points are highly associated. CFB in VAS pain score at 2 weeks was predictive of response at both 6 and 12 weeks. CFB in VAS pain score at 6 weeks is also predictive and almost identical to CFB in VAS pain score at 12 weeks. This is in accordance with individual patient-level responder analysis of etoricoxib, celecoxib, naproxen and ibuprofen over 2–12 weeks, where the proportion of patients achieving various levels of response was consistent at 2, 4, 8 and 12 weeks [22].

Earlier, Bingham and colleagues demonstrated a similar predictive effect of early response to NSAIDs in predicting later response in a pooled analysis of two identical 26-week studies testing etoricoxib, celecoxib and placebo in patients with OA of the hip and knee. With active treatment, around 75 % of patients who were responders at 2 weeks were also responders at 12 weeks [16]. This is also the case in fibromyalgia [17] and acute pain in individual patient-level analyses [18]. In these examples, the converse is also true: lack of early response indicates that later response is very unlikely. The situation may be different for the anti-depressant duloxetine in several chronic pain states [23].

The finding that early response is predictive of later response is important in a number of ways. In clinical practice, for example, it can be used to consider ‘stopping rules’, whereby the failure to achieve a certain level of pain relief by 2 or 6 weeks with one NSAID would mean that the therapy is reviewed, the dose is escalated or a switch is made to another analgesic. Together with the knowledge that analgesics provide good pain relief in only a portion of patients [23], this can change the way guidance is formulated. For example, an early opportunity to assess efficacy and switch in the case of non-response might be built into care pathways in the future. This opportunity may not be available in current usual care, where patients may discuss therapeutic responses many months after treatments have been initiated. Early switching forms part of the guidance for neuropathic pain in England and Wales.

These ideas can also inform appropriate treatment duration in designing a clinical trial (with consideration of both tolerability and efficacy). For example, studies of efficacy could be shorter, perhaps 6 instead of 12 weeks. But studies of harm, which are typically longer and often larger, might be considered unethical if they included a large proportion of patients who were at risk but had no benefit; studies of harm might have to have quite different designs, perhaps based on cohorts of only those patients who actually benefit. However, the actual design can build in aspects of this knowledge, either in pragmatic trials of switching therapy in the face of non-response, designs for which have been proposed [23], or in the greater use of enriched enrolment randomised withdrawal designs [24].

Limitations of this study should be borne in mind when interpreting the results. The limitations related to availability of data and the potential for within-study bias and publication bias have been extensively discussed elsewhere [20]. Our analyses are directed to within-trial comparisons, where issues relating to quality and availability of data or use of different imputation methods for missing data are minimised. There is no reason to suspect any differential effect of publication bias, as the results of the original searches were cross-checked against the results of the Coxib and traditional NSAID Trialists’ Collaboration study [25]. Furthermore, the analyses presented herein are based on aggregated data; thus, and while there is a theoretical risk of ecological fallacy (i.e., results not translatable on the individual patient level), there is existing evidence from individual patient-level analyses in OA that this is not the case [16]. One final comment is that we have concentrated only on patient-reported pain; in few studies have researchers reported markers of inflammation, and none commented on links between inflammation and pain in the timing of any changes on average or in individual patients.

The predictive ability of the models was assessed by examining the discrepancies between observed and predicted values and the standardised residuals. In Additional file 1, it can be seen that all models perform well.

## Conclusions

For patients with OA and RA, early analgesic response measured by VAS for pain beyond 2 weeks of treatment with a particular NSAID is likely to be predictive of response at 12 weeks. Failure to achieve desired pain relief after 2 weeks of treatment should trigger reassessment of dosage and/or choice of analgesic.

## Additional files

Additional file 1: Additional results. **Figure S1.** N-weighted random intercept model: CFB 2 weeks vs. CFB 6 weeks observed vs. predicted values. **Figure S2.** Precision-weighted random intercept model: CFB 2 weeks vs. CFB 6 weeks observed vs. predicted values and standardised residuals. **Figure S3.** N-weighted random intercept model: CFB 2 weeks vs. CFB 12 weeks observed vs. predicted values and standardised residuals. **Figure S4.** Precision-weighted random intercept model: CFB 2 weeks vs. CFB 12 weeks observed vs. predicted values and standardised residuals. **Figure S5.** N-weighted random intercept model: CFB 6 weeks vs. CFB 12 weeks observed vs. predicted values and standardised residuals. **Figure S6.** Precision-weighted random intercept model: CFB 6 weeks vs. CFB 12 weeks observed vs. predicted values and standardised residuals. (PDF 969 kb) Additional file 2: Study design. (PDF 161 kb) Additional file 3: Patient characteristics. (PDF 321 kb) Additional file 4: Input data tables. (PDF 368 kb)

## Abbreviations

AIC: Akaike information criterion
CFB: change from baseline
CI: confidence interval
COXIB: cyclooxygenase 2 inhibitor
NSAID: non-steroidal anti-inflammatory drug
OA: osteoarthritis
RA: rheumatoid arthritis
RCT: randomised controlled trial
SE: standard error
VAS: visual analogue scale
