# Supplementary material for: High correlation of VAS pain scores after 2 and 6 weeks of treatment with VAS pain scores at 12 weeks in randomised controlled trials in rheumatoid arthritis and osteoarthritis: meta-analysis and implications
Source: Arthritis Res Ther. 2016 Mar 31;18:73. doi: 10.1186/s13075-016-0972-7 (PMC4818534; doi:10.1186/s13075-016-0972-7)

Figure S1: N weighted - Random Intercept model: CFB 2 vs. CFB 6 weeks observed vs predicted values

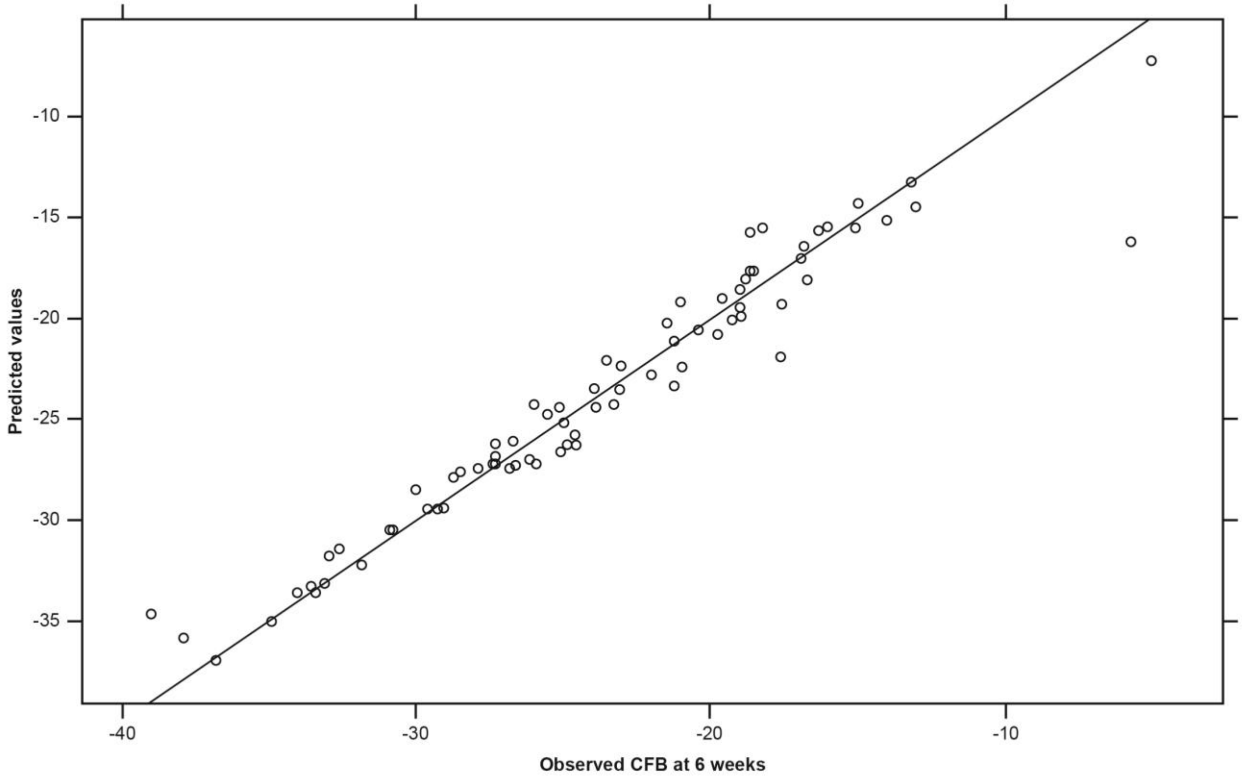

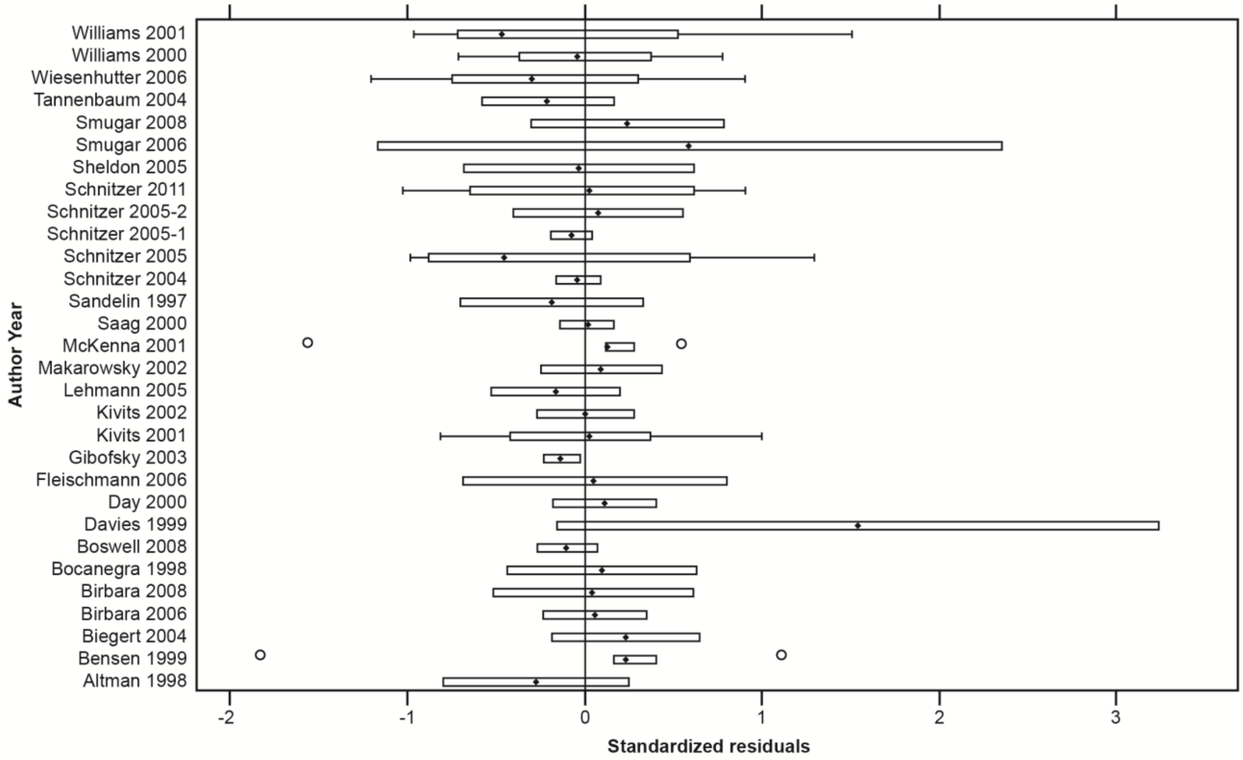

Figure S2: Precision weighted - Random Intercept model: CFB 2 vs. CFB 6 weeks observed vs predicted values and standardized residuals

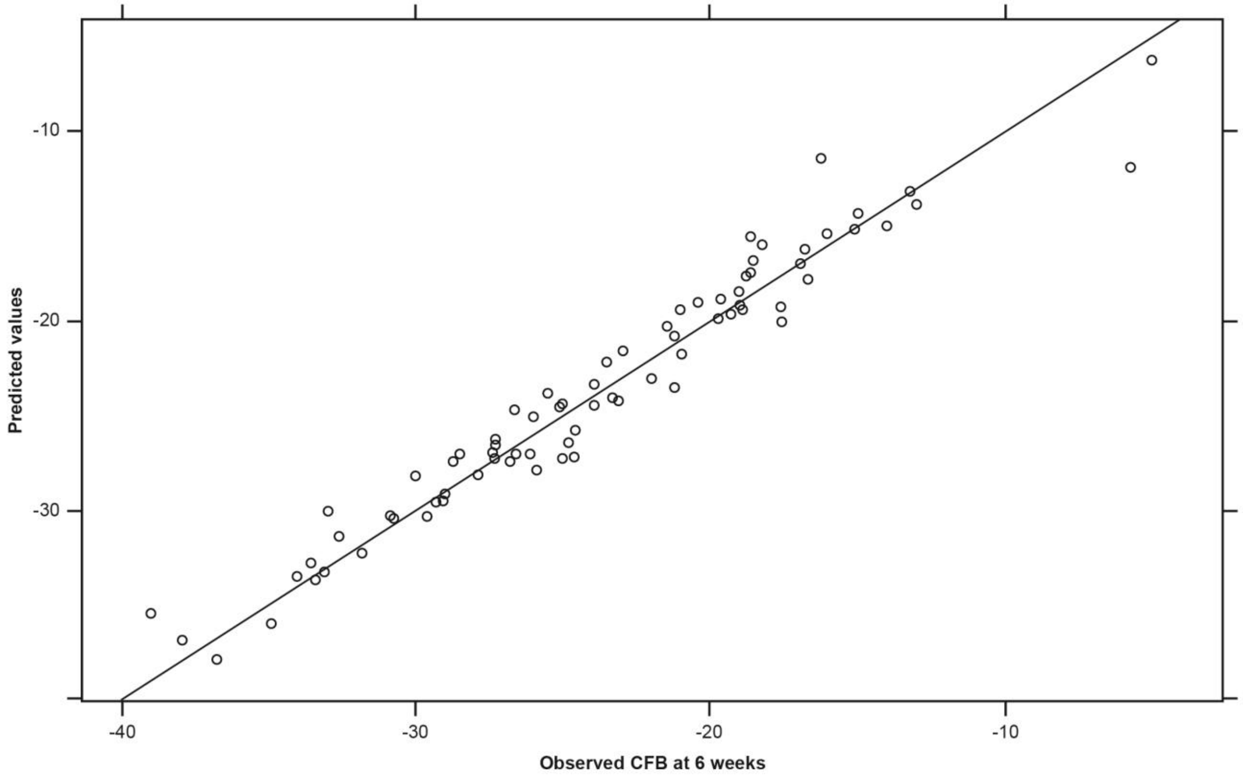

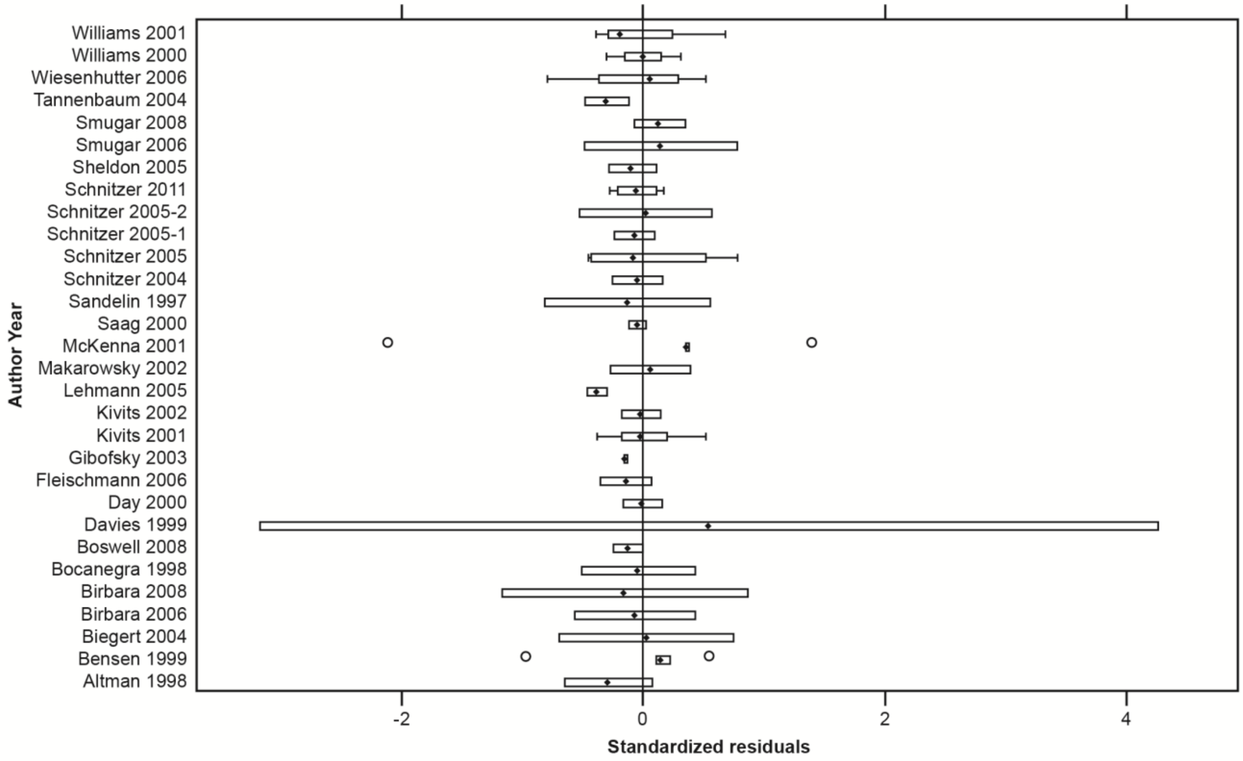

Figure S3: N weighted - Random Intercept model: CFB 2 vs. CFB 12 weeks observed vs predicted values and standardized residuals

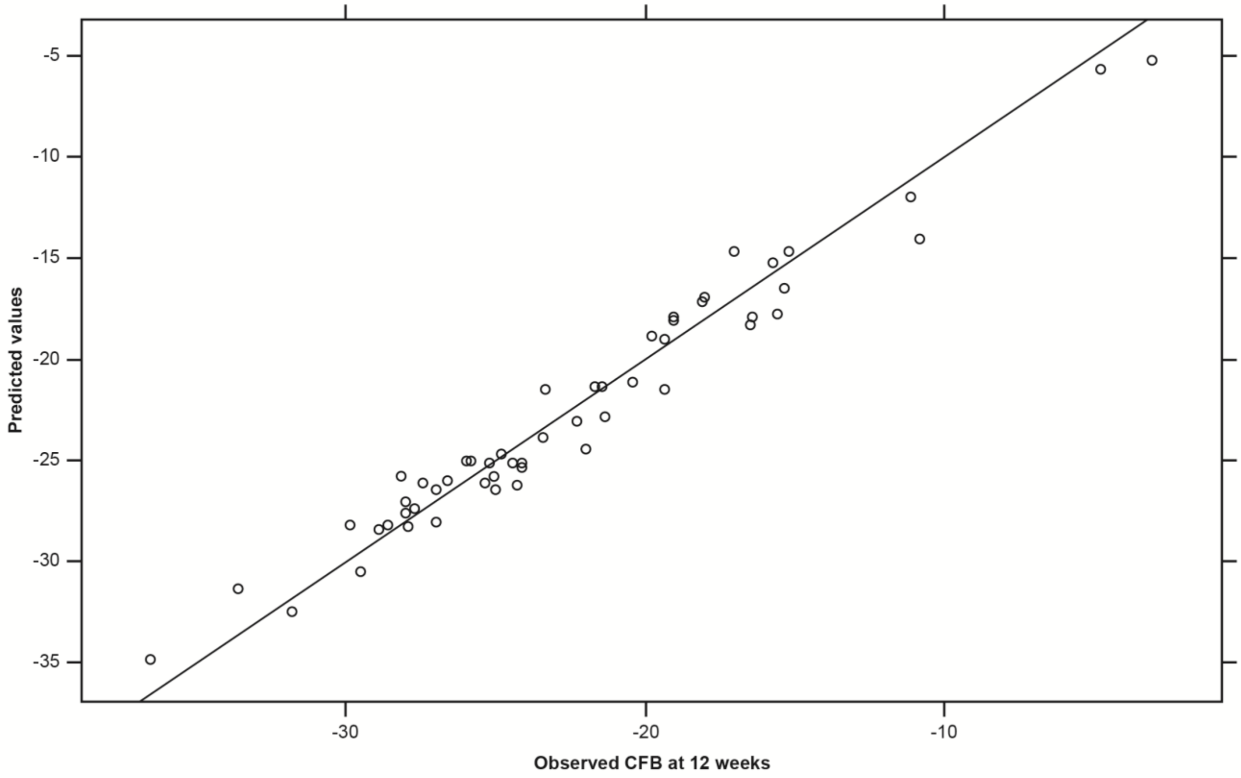

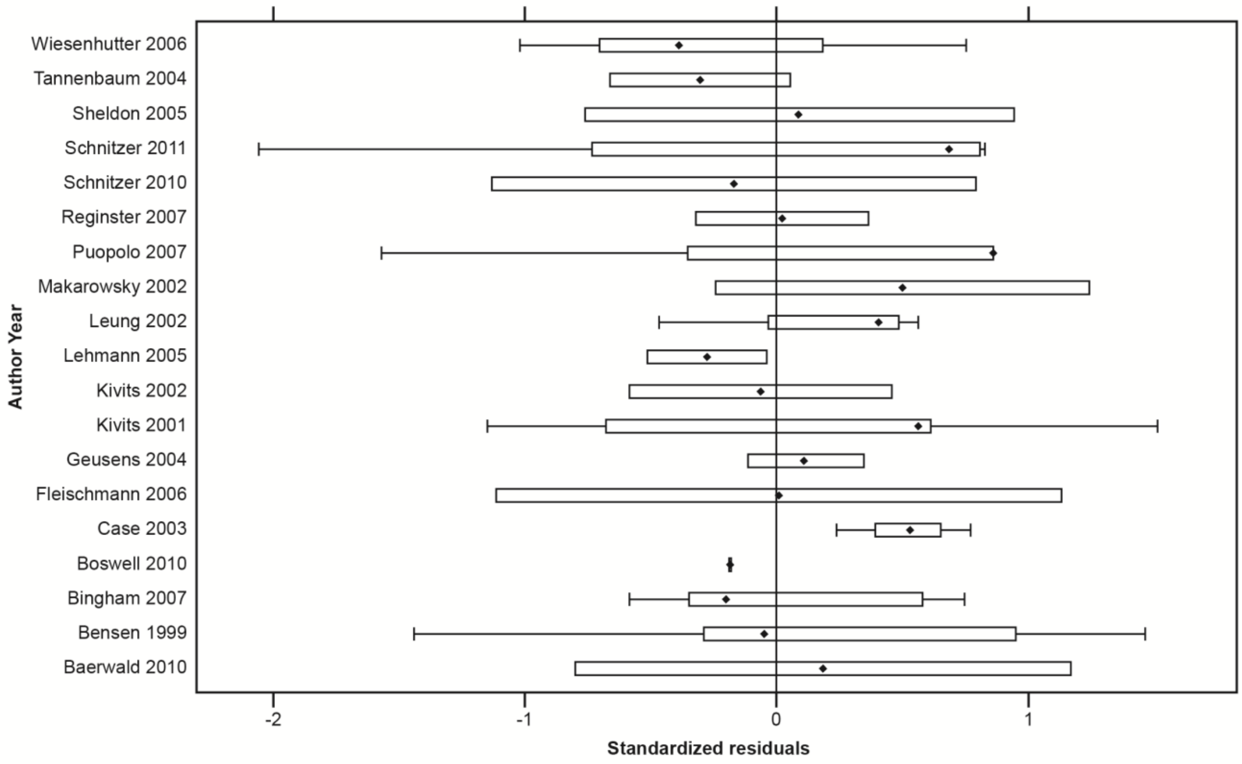

Figure S4: Precision weighted - Random Intercept model: CFB 2 vs. CFB 12 weeks observed vs predicted values and standardized residuals

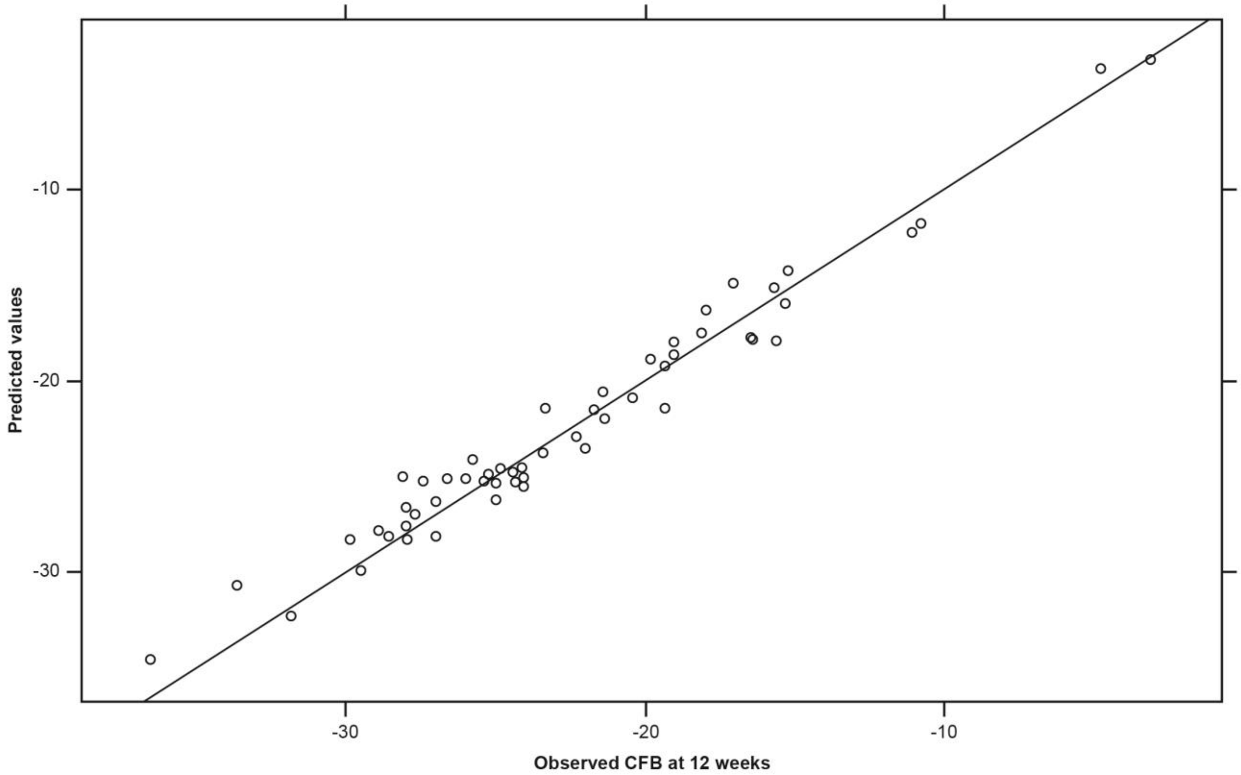

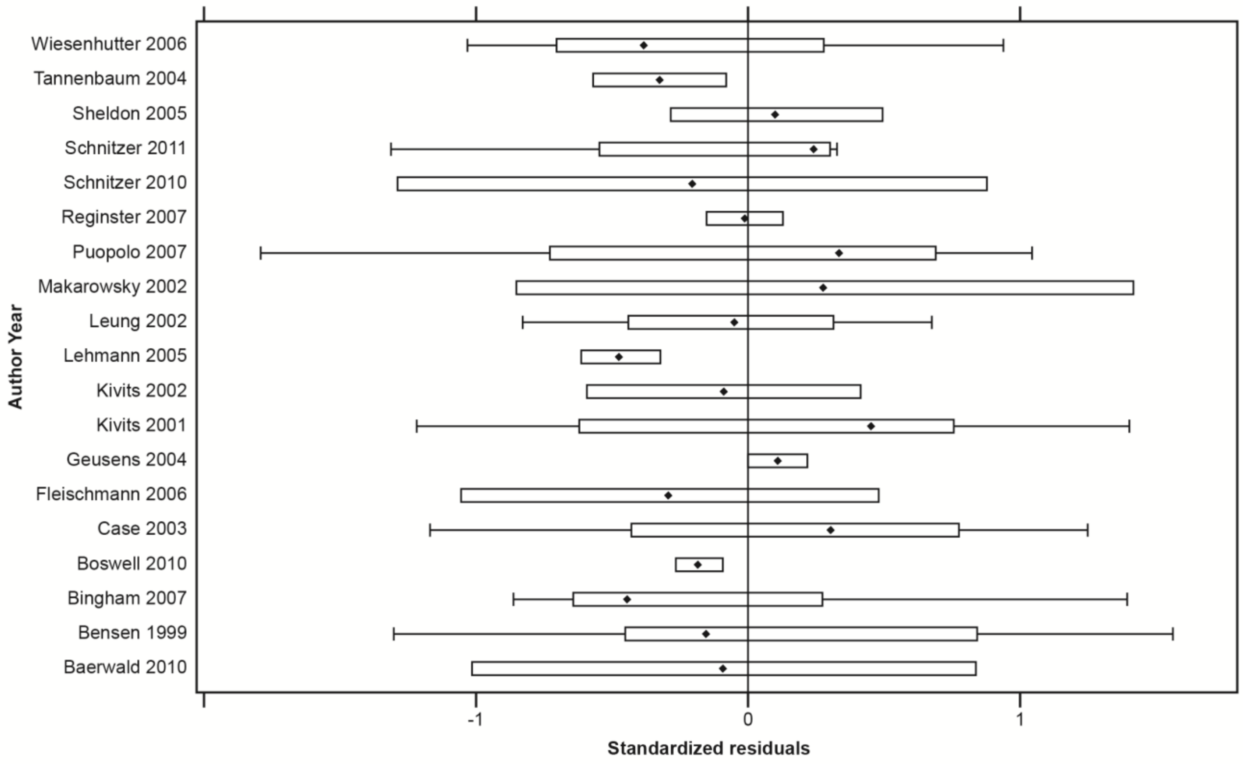

Figure S5: N weighted - Random Intercept model: CFB 6 vs. CFB 12 weeks observed vs predicted values and standardized residuals

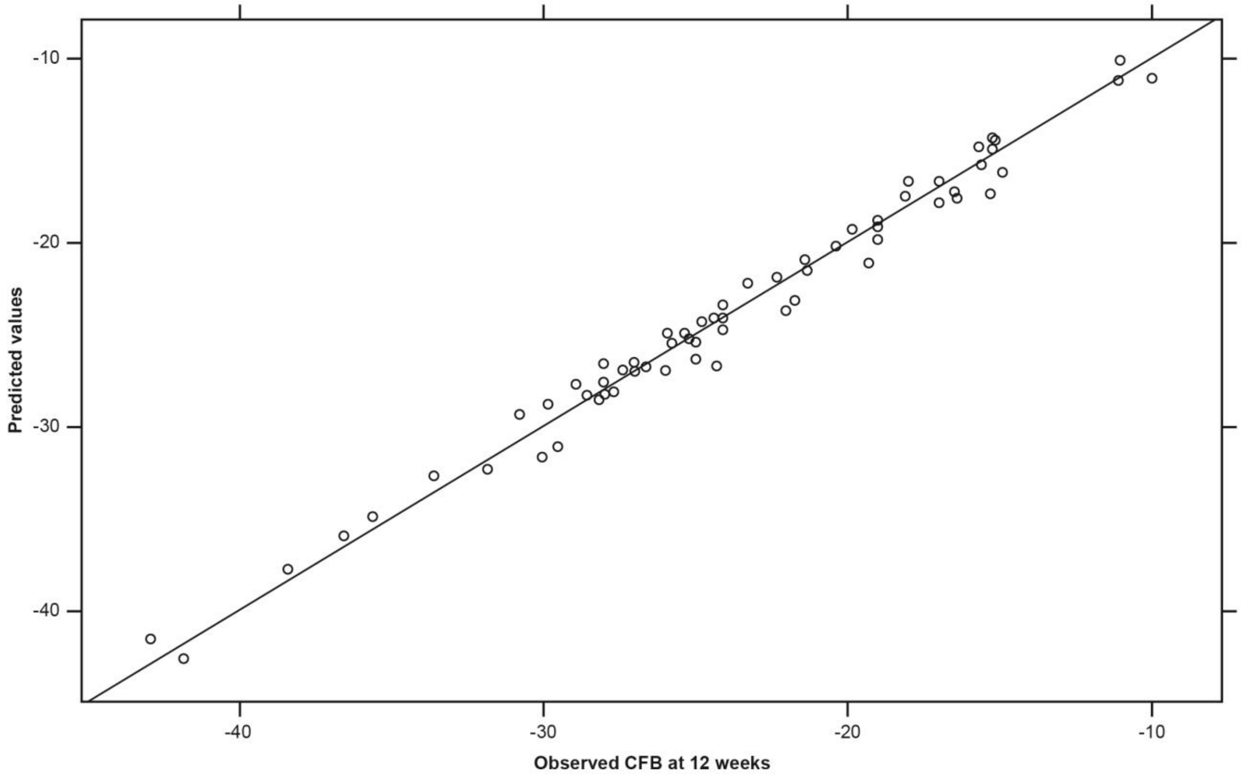

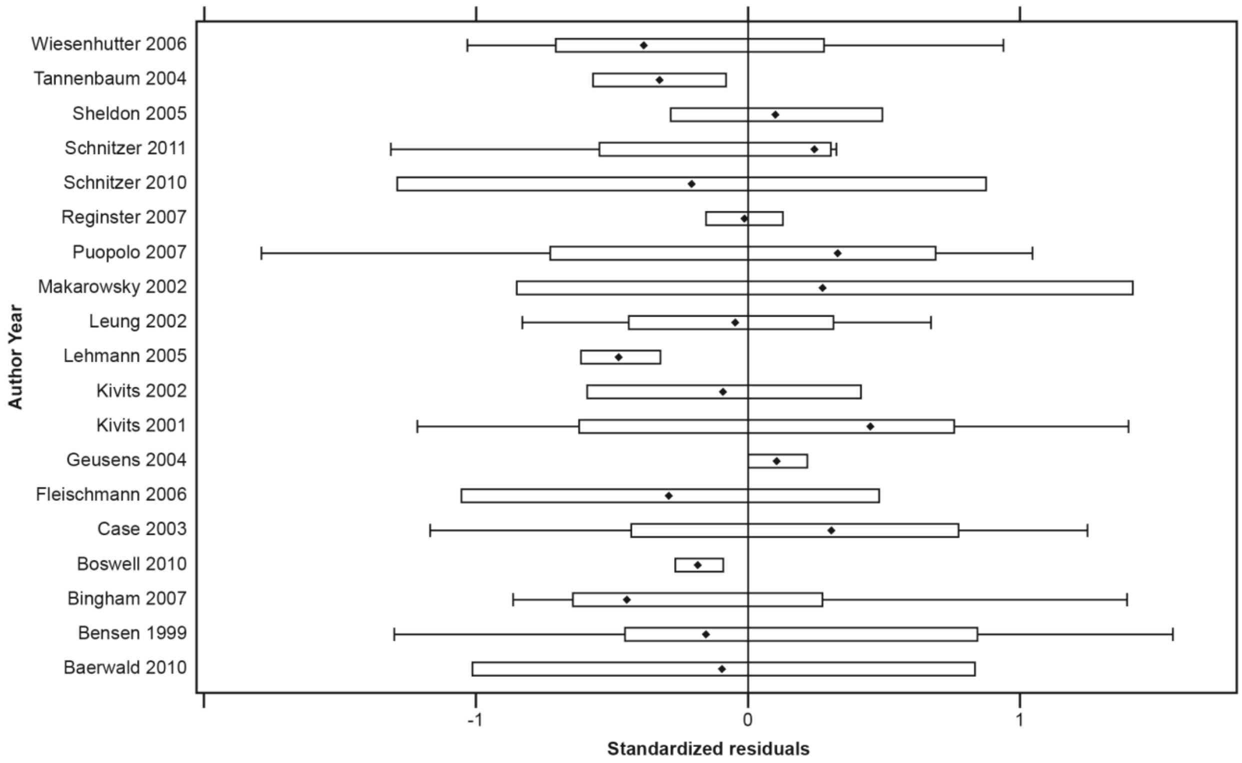

Figure S6: Precision weighted - Random Intercept model: CFB 6 vs. CFB 12 weeks observed vs predicted values and standardized residuals

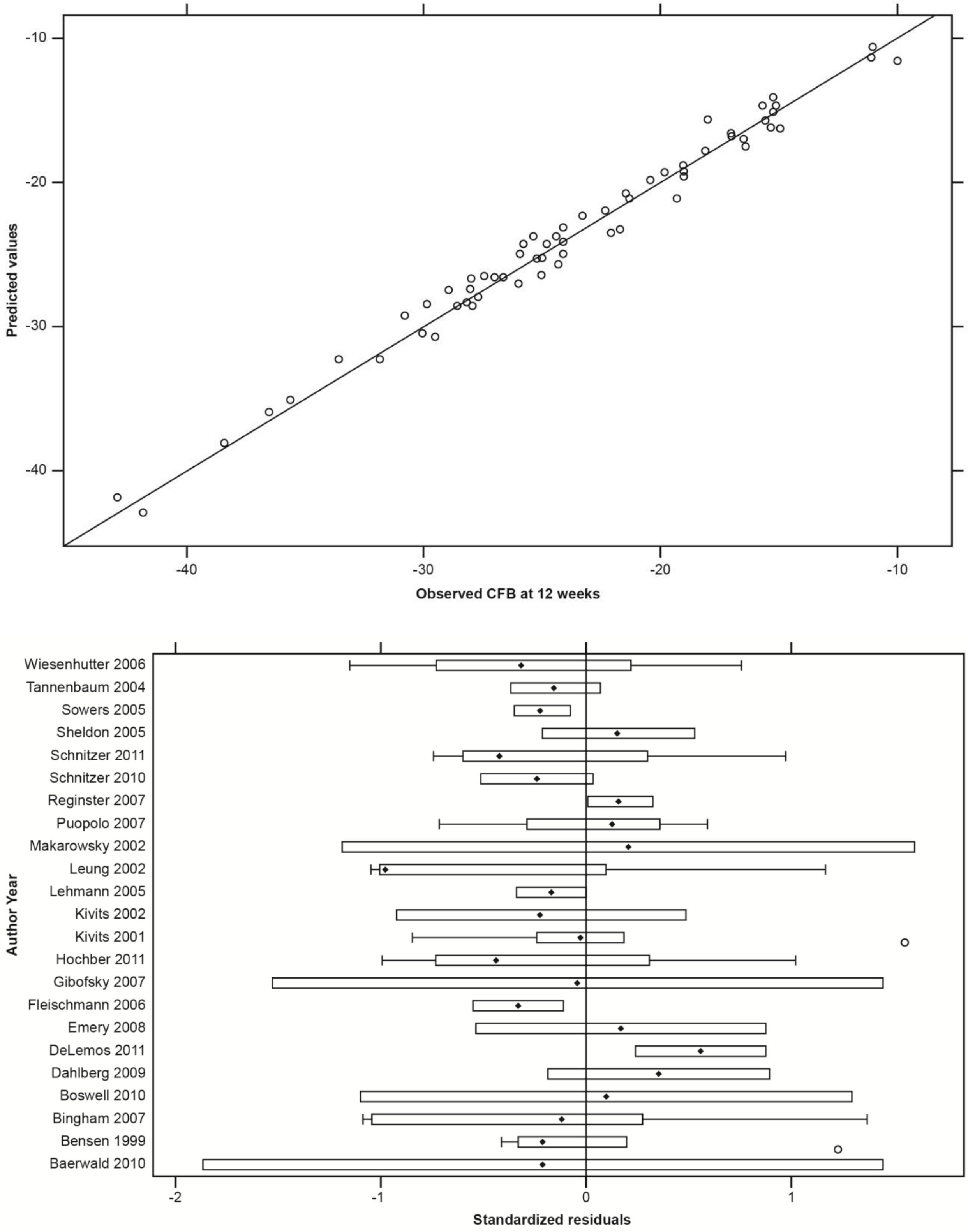

Supplement: Additional file 1: — Additional results. Figure S1. N-weighted random intercept model: CFB 2 weeks vs. CFB 6 weeks observed vs. predicted values. Figure S2. Precision-weighted random intercept model: CFB 2 weeks vs. CFB 6 weeks observed vs. predicted values and standardised residuals. Figure S3. N-weighted random intercept model: CFB 2 weeks vs. CFB 12 weeks observed vs. predicted values and standardised residuals. Figure S4. Precision-weighted random intercept model: CFB 2 weeks vs. CFB 12 weeks observed vs. predicted values and standardised residuals. Figure S5. N-weighted random intercept model: CFB 6 weeks vs. CFB 12 weeks observed vs. predicted values and standardised residuals. Figure S6. Precision-weighted random intercept model: CFB 6 weeks vs. CFB 12 weeks observed vs. predicted values and standardised residuals. (PDF 969 kb) [file 13075_2016_972_MOESM1_ESM.pdf]
