# Supplementary material for: High correlation of VAS pain scores after 2 and 6 weeks of treatment with VAS pain scores at 12 weeks in randomised controlled trials in rheumatoid arthritis and osteoarthritis: meta-analysis and implications
Source: Arthritis Res Ther. 2016 Mar 31;18:73. doi: 10.1186/s13075-016-0972-7 (PMC4818534; doi:10.1186/s13075-016-0972-7)
Supplement: Additional file 2: — Study design. (PDF 161 kb) [file 13075_2016_972_MOESM2_ESM.pdf]

Author Year	Trial name	Study design	Compared interventions	Trial duration in weeks	Patient population	Jadad score out of 5
Gibofsky 2003	NR	RCT, DB, PC, MC	Celecoxib, 200mg/d, oral, OD	6	OA	5
			Placebo			
Reginster 2007	NR	RCT, PC, AC, DB, MC, DD, PG,	Etoricoxib, 60mg/d, oral, OD	12 PC - 40 AC (*1)	OA	5
			Naproxen, 1000mg/d, oral, BID			
Bingham 2007	Protocol 076 (Study 1)	RCT, PC, DB, MC, DD	Etoricoxib, 30mg/d, oral, OD	12 PC - 14 AC (*2)	OA	3
			Celecoxib, 200mg/d, oral, OD			
			Placebo			
	Protocol 077 (Study 2)	RCT, PC, DB, MC, DD	Etoricoxib, 30mg/d, oral, OD	12 PC - 14 AC (*2)	OA	
			Celecoxib, 200mg/d, oral, OD			
			Placebo			
Baerwald 2010	NR	RCT, DB, PG, PC, AC, MC	Naproxen, 1000mg/d, oral, BID	13	OA	3
			Placebo			
Day 2000	NR	RCT, DB, MC	Ibuprofen, 2400mg/d, oral, TID	6	OA	5
			Placebo			
Schnitzer 2005a	NR	RCT, DB, PG, PC	Naproxen, 1000mg/d, oral, BID	6	OA	5
			Placebo			
Schnitzer 2005b	VACT-1 and VACT-2 (Protocols 106 and 150)	RCT, DB, AC, MC	Celecoxib, 200 mg/d, oral, OD	6	OA	5
			Acetaminophen, 4000 mg/d, oral, QID			
Sowers 2005	NR	RCT, DB, AC, MC	Celecoxib 200mg/d, oral, OD	12	OA	3
			Naproxen 1000mg/d, oral, BID			
Schnitzer 2004b	NR	RCT, PC, DB, DD, PG, MC	Diclofenac, 150mg/d, oral, BID	4	OA	3

			Placebo			
Tannenbaum 2004	NR	RCT, PC, AC, DB, MC	Celecoxib, 200mg/d, oral, OD	13	OA	4
			Placebo			
Schnitzer 2010	NR	RCT, AC, PC, DB, MC, PG	Naproxen, 1000mg/d, oral, BID	13	OA	3
			Placebo			
Dahlberg 2009	NR	RCT, DB, MC	Celecoxib, 200mg/d, oral, OD	52	OA	5
			Diclofenac, 100mg/d, oral, BID			
Schnitzer 2011b	NR	RCT, PC, DB, MC	Naproxen, 1000 mg/d, oral, BID	53	OA	3
			Placebo and after 13 weeks, naproxcinod 375mg/d, BID			
			Placebo and after 13 weeks, naproxcinod 750mg/d, BID			
Schnitzer 2011a	NCT00154219	RCT, AC, PC, DB, MC	Celecoxib, 200mg/d, oral, OD	13	OA	5
			Placebo			
Smugar 2006	Study 1 (Protocol 112)	RCT, AC, PC, DB, PG, MC	Celecoxib, 200mg/d, oral, OD	6	OA	2
			Placebo			
	Study 2 (Protocol 116)	RCT, AC, PC, DB, PG, MC	Celecoxib, 200mg/d, oral, OD	6	OA	
			Placebo			
Emery 2008	NR	RCT, DB, DD, PG	Celecoxib, 200mg/d, oral, OD	12	OA (requiring joint replacement)	5
			Diclofenac, 150mg/d, oral, TID			
Gibofsky 2007	NR	RCT, MC, PC, DB	Naproxen 1000mg/d, oral, BID	12	RA	4
			Placebo			
McKenna 2001a	NR	RCT, DB, PC, MC, PG	Celecoxib, 200mg/d, oral, BID	6	OA	3

			Diclofenac 150mg/d, oral, TID			
			Placebo			
McKenna 2001b	NR	RCT, DB, PC, MC	Celecoxib, 200mg/d, oral, OD	6	OA	4
			Placebo			
Williams 2000	NR	RCT, DB, PC, PG, MC	Celecoxib, 200mg/d, oral, BID	6	OA	4
			Celecoxib, 200mg/d, oral, OD			
			Placebo			
Bocanegra 1998	Arthrotec Osteoarthritis Study Group	RCT, DB, PC, PG, MC	Diclofenac sodium, 150mg/d, oral, BID	6	OA	3
			Placebo			
Wiesenhutter 2005	Protocol 071	RCT, DB, PC, AC, MC	Etoricoxib, 30mg/d, oral, OD	12	OA	4
			Ibuprofen, 2400mg/d, oral, TID			
			Placebo			
Sheldon 2005	NR	RCT, PC, DB, DD, PG	Celecoxib, 200mg/d, oral, OD	13	OA	4
			Placebo			
Biegert 2004	NR	RCT, DB, PC, MC, PG	Diclofenac, 100mg/d, oral, BID, enteric coated	6	OA	5
			Placebo			
Zhao 1999/Bensen 1999	NR	RCT, DB, MC, PC, PG	Celecoxib, 100mg/d, oral, BID	12	OA	3
			Celecoxib, 200mg/d, oral, BID			
			Celecoxib, 400mg/d, oral, BID			
			Naproxen, 1000mg/d, oral, BID			
			Placebo			
DeLemos 2011	NR	RCT, PC, DB, MC	Celecoxib 200 mg/d, oral, OD	12	OA	4
			Placebo			

Fleischmann 2005	NR	RCT, PC, DB, DD, MC, PG	Celecoxib, 200mg/d, oral, OD	13	OA	5
			Placebo			
Davies 1999	NR	RCT, PC, DB, PG, MC	Ibuprofen, 2400mg/d, oral, TID	4	OA	2
			Placebo			
Lehmann 2005	NR	RCT, PC, DB, DD, MC	Celecoxib, 200mg/d, oral, OD	13	OA	5
			Placebo			
Altman 1998	NR	RCT, PC, DB, DD, PG, MC	Naproxen, 1000mg/d, oral, BID	26	OA	4
			Placebo			
Puopolo 2007	NR	RCT, PC, AC, DB, MC	Etoricoxib, 30mg/d, oral, OD	12	OA	5
			Ibuprofen, 2400mg/d, oral, TID			
			Placebo			
Hochberg 2011/Cryer 2011	Study 307 (PN400-307 - NCT00664560)	RCT, PC, DB, PG, MC	Celecoxib, 200mg/d, oral, OD	12	OA	5
			Placebo			
	Study 309 (PN400-309 - NCT00665431)	RCT, PC, DB, PG, MC	Celecoxib, 200 mg/d, oral, OD	12	OA	
			Placebo			
Williams 2001	NR	RCT, PC, DB, PG, MC	Celecoxib, 200mg/d, oral, BID	6	OA	4
			Celecoxib, 200mg/d, oral, OD			
			Placebo			
Leung 2002	NR	RCT, PC, AC DB, MC	Etoricoxib, 60mg, oral, OD	12	OA	5
			Naproxen, 1000mg/d, oral, BID			
			Placebo			
Makarowski 2002	NR	RCT, PC, DB, MC, PG	Naproxen, 100mg/d, oral, BID	12	OA	4
			Placebo			

Kivitz 2001	NR	RCT, PC, DB, MC, PG	Celecoxib, 100mg/d, oral, BID	12	OA	4
			Celecoxib, 200mg/d, oral, BID			
			Celecoxib, 400mg/d, oral, BID			
			Naproxen, 1000mg/d, oral, BID			
			Placebo			
Boswell 2008	Study A (GSK protocol CXA20005)	RCT, PC, AC, DB, MC, PG	Celecoxib, 200mg/d, oral, OD	6	OA	3
			Placebo			
	Study B (GSK protocol CXA30007)		Celecoxib, 200mg/d, oral, OD			
			Placebo			
Sandelin 1997	NR	RCT, PC, DB, MC	Diclofenac, 100mg/d, oral, BID, combined with placebo gel, 9g/d, TID	4	OA	5
			Placebo gel and tablet			
Birbara 2006	NR (Study 1)	RCT, PC, DB, MC, PG	Celecoxib, 200mg/d, oral, OD	6	OA	5
			Placebo			
	NR (Study 2)	RCT, PC, DB, MC, PG	Celecoxib, 200mg/d, oral, OD	6	OA	
			Placebo			
Case 2003	NR	RCT, DB, PC, SC	Diclofenac sodium, 150 mg/d, oral, BID	12	OA	4
			Acetaminophen, 4000 mg/d, oral, QID			
			Placebo			
Schnitzer 2005c-1	NR	RCT, PC, DB, MC	Diclofenac, 150 mg/d, oral, BID	4	OA	4
			Placebo			
Schnitzer 2005c-2	NR	RCT, PC, DB, MC	Diclofenac, 150 mg/d, oral, BID	4	RA	
			Placebo			

Kivitz 2002	NR	RCT, DB, PC, MC	Naproxen, 1000 mg/d, oral, BID	12	OA	3
			Placebo			
Geusens 2004	NR	RCT, DB, DD, PC, AC, PG, MC	Naproxen, 1000mg/d, oral, BID	26	RA	4
			Placebo			
Saag 2000	NR	RCT, DB, PC, AC,	Ibuprofen, 2400mg/d, oral, TID	6	OA	5
			Placebo			
