# Supplementary material for: High correlation of VAS pain scores after 2 and 6 weeks of treatment with VAS pain scores at 12 weeks in randomised controlled trials in rheumatoid arthritis and osteoarthritis: meta-analysis and implications
Source: Arthritis Res Ther. 2016 Mar 31;18:73. doi: 10.1186/s13075-016-0972-7 (PMC4818534; doi:10.1186/s13075-016-0972-7)
Supplement: Additional file 3: — Patient characteristics. (PDF 321 kb) [file 13075_2016_972_MOESM3_ESM.pdf]

Author Year	Trial name	Compared interventions	N [randomized patients]	Age in years [mean]	Gender [% Male]	Duration of disease in years [Mean]	Hypertension [%]	Current smoker [%]	History of GI ulcer [%]	Gastro-protective agent use allowed [yes/no]	Aspirin use allowed [yes/no]	Rescue medication use allowed [yes/no]
Gibofsky 2003	NR	Celecoxib, 200mg/d, oral, OD	189	62	31%	9	NR	NR	6%	No	Yes	Yes
		Placebo	98	63	35%	8			6%			
Reginster 2007	NR	Etoricoxib, 60mg/d, oral, OD	446	63	28%	NR	NR	NR	NR	Yes	Yes	Yes
		Naproxen, 1000mg/d, oral, BID	439	63	29%							
Bingham 2007	Protocol 076 (Study 1)	Etoricoxib, 30mg/d, oral, OD	231	62	34%	NR	NR	NR	NR	NR	Yes	Yes
		Celecoxib, 200mg/d, oral, OD	241	63	30%							
		Placebo	127	63	35%							
	Protocol 077 (Study 2)	Etoricoxib, 30mg/d, oral, OD	244	62	30%	NR	NR	NR	NR	NR	Yes	Yes
		Celecoxib, 200mg/d, oral, OD	247	62	38%							
		Placebo	117	61	35%							
Baerwald 2010	NR	Naproxen, 1000mg/d, oral, BID	156	62	32%	NR	48%	NR	EC	NR	Yes	Yes
		Placebo	331	63	37%		54%					
Day 2000	NR	Ibuprofen, 2400mg/d, oral, TID	249	64	22%	9	NR	NR	EC	NR	No	Yes
		Placebo	74	63	15%	9			EC			
Schnitzer 2005a	NR	Naproxen, 1000mg/d, oral, BID	117	60	39%	NR	NR	NR	EC	NR	NR	Yes
		Placebo	104	61	38%							

Schnitzer 2005b	VACT-1 and VACT-2 (Protocols 106 and 150)	Celecoxib, 200 mg/d, oral, OD	523	61	32%	NR	43%	NR	NR	NR	No	NR
		Acetaminophen, 4000 mg/d, oral, QID	269	62	34%							
Sowers 2005	NR	Celecoxib 200mg/d, oral, OD	136	62	38%	NR	100%	NR	NR	NR	Yes	NR
		Naproxen 1000mg/d, oral, BID	130	64	40%		100%					
Schnitzer 2004b	NR	Diclofenac, 150mg/d, oral, BID	94	60	32%	6	NR	NR	EC	No	NR	Yes
		Placebo	97	62	33%	8						
Tannenbaum 2004	NR	Celecoxib, 200mg/d, oral, OD	481	64	31%	5 (*1)	NR	NR	NR	NR	Yes	Yes
		Placebo	243	65	33%	4 (*1)						
Schnitzer 2010	NR	Naproxen, 1000mg/d, oral, BID	227	61	32%	NR	48%	NR	EC	NR	Yes	Yes
		Placebo	221	61	28%		51%					
Dahlberg 2009	NR	Celecoxib, 200mg/d, oral, OD	463	71	32%	NR	NR	NR	EC	No	Yes	Yes
		Diclofenac, 100mg/d, oral, BID	462	71	31%							
Schnitzer 2011b	NR	Naproxen, 1000 mg/d, oral, BID	254	60	30%	NR	51%	NR	EC	NR	Yes	Yes
		Placebo and after 13 weeks, naproxcinod 375mg/d, BID	123	60	30%		56%					
		Placebo and after 13 weeks, naproxcinod 750mg/d, BID	134	60	25%		42%					
Schnitzer 2011a	NCT00154219	Celecoxib, 200mg/d, oral, OD	419	62	39%	4	NR	NR	EC	NR	NR	Yes
		Placebo	416	61	39%	4						
Smugar 2006	Study 1	Celecoxib, 200mg/d, oral, OD	456	62	33%	NR	NR	NR	NR	No	Yes	Yes

		Placebo	150	62	31%							
	Study 2	Celecoxib, 200mg/d, oral, OD	460	62	34%	NR	NR	NR	NR	No	Yes	Yes
		Placebo	151	63	33%							
Emery 2008	NR	Celecoxib, 200mg/d, oral, OD	69	63	52%	NR	NR	NR	EC	No	Yes	Yes
		Diclofenac, 150mg/d, oral, TID	72	65	56%							
Gibofsky 2007	NR	Naproxen 1000mg/d, oral, BID	167	57	29%	10	NR	NR	EC	NR	Yes	Yes
		Placebo	171	56	16%	12						
McKenna 2001a	NR	Celecoxib, 200mg/d, oral, BID	201	62	32%	8	NR	NR	8%	NR	Yes	NR
		Diclofenac 150mg/d, oral, TID	199	63	28%	9			6%			
		Placebo	200	60	34%	9			9%			
McKenna 2001b	NR	Celecoxib, 200mg/d, oral, OD	63	62	33%	11	NR	NR	10%	No	Yes	NR
		Placebo	60	63	25%	12			12%			
Williams 2000	NR	Celecoxib, 200mg/d, oral, BID	232	63	33%	9	NR	NR	EC	NR	Yes	Yes
		Celecoxib, 200mg/d, oral, OD	231	63	33%	9						
		Placebo	223	63	34%	9						
Bocanegra 1998	Arthrotec Osteoarthritis Study Group	Diclofenac sodium, 150mg/d, oral, BID	154	63	29%	12	NR	NR	100%	NR	NR	NR
		Placebo	91	62	32%	11						
Wiesenhutter 2005	Protocol 071	Etoricoxib, 30mg/d, oral, OD	214	63	30%	8	41%	NR	NR	Yes	Yes	NR
		Ibuprofen, 2400mg/d, oral, TID	210	61	30%	8						

		Placebo	104	60	28%	7						
Sheldon 2005	NR	Celecoxib, 200mg/d, oral, OD	393	60	37%	7	NR	NR	EC	Yes	Yes	Yes
		Placebo	382	61	39%	7						
Biegert 2004	NR	Diclofenac, 100mg/d, oral, BID, enteric coated	43	61	35%	9	NR	NR	EC	NR	Yes	No
		Placebo	41	62	39%	11						
Zhao 1999/Bensen 1999	NR	Celecoxib, 100mg/d, oral, BID	203	62	31%	9	NR	NR	EC	NR	Yes	Yes
		Celecoxib, 200mg/d, oral, BID	197	62	27%	10						
		Celecoxib, 400mg/d, oral, BID	202	63	28%	9						
		Naproxen, 1000mg/d, oral, BID	198	62	29%	10						
		Placebo	204	62	25%	10						
DeLemos 2011	NR	Celecoxib 200 mg/d, oral, OD	203	60	35%	8	NR	NR	EC	NR	Yes	NR
		Placebo	202	59	32%	8						
Fleischmann 2005	NR	Celecoxib, 200mg/d, oral, OD	444	61	33%	7	NR	NR	NR	No	Yes	Yes
		Placebo	231	62	34%	7						
Davies 1999	NR	Ibuprofen, 2400mg/d, oral, TID	54	61	37%	8	NR	NR	EC	NR	NR	Yes
		Placebo	50	62	36%	8						
Lehmann 2005	NR	Celecoxib, 200mg/d, oral, OD	420	63	32%	4	NR	NR	EC	Yes	Yes	Yes
		Placebo	424	62	28%	4						
Altman 1998	NR	Naproxen, 1000mg/d, oral, BID	113	63	43%	NR	NR	NR	NR	NR	NR	Yes
		Placebo	115	65	47%							
Puopolo 2007	NR	Etoricoxib, 30mg/d, oral,	224	62	22%	7	46%	NR	4%	Yes	Yes	Yes

		OD										
		Ibuprofen, 2400mg/d, oral, TID	213	62	26%	7						
		Placebo	111	64	24%	7						
Hochberg 2011/Cryer 2011	Study 307 (PN400-307)	Celecoxib, 200mg/d, oral, OD	242	62	39%	NR	NR	14%	NR	No	Yes	Yes
		Placebo	124	62	34%			18%				
	Study 309 (PN400-309)	Celecoxib, 200 mg/d, oral, OD	244	62	37%	NR	NR	17%	NR	No	Yes	Yes
		Placebo	122	62	37%			9%				
Williams 2001	NR	Celecoxib, 200mg/d, oral, BID	243	62	31%	10	NR	NR	EC	NR	Yes	Yes
		Celecoxib, 200mg/d, oral, OD	231	61	31%	9						
		Placebo	244	61	27%	10						
Leung 2002	NR	Etoricoxib, 60mg, oral, OD	224	63	23%	6	NR	NR	NR	Yes	NR	Yes
		Naproxen, 1000mg/d, oral, BID	221	63	22%	6						
		Placebo	56	64	18%	6						
Makarowski 2002	NR	Naproxen, 100mg/d, oral, BID	118	63	31%	5	NR	NR	9%	NR	Yes	NR
		Placebo	118	62	31%	6			11%			
Kivitz 2001	NR	Celecoxib, 100mg/d, oral, BID	216	62	35%	7	NR	NR	9-18%	NR	Yes	Yes
		Celecoxib, 200mg/d, oral, BID	207	62	35%	7						
		Celecoxib, 400mg/d, oral, BID	213	61	33%	7						
		Naproxen, 1000mg/d, oral, BID	207	64	34%	7						
		Placebo	218	64	33%	8						
Boswell 2008	Study A (GSK protocol	Celecoxib, 200mg/d, oral,	109	63,3	36%	7	NR	NR	EC	Yes	NR	Yes

	CXA20005)	OD										
		Placebo	107	63,5	29%	6						
	Study B (GSK protocol CXA30007)	Celecoxib, 200mg/d, oral, OD	185	59,7	33%	9	NR	NR	EC	Yes	No	Yes
		Placebo	186	60,5	27%	8						
Sandelin 1997	NR	Diclofenac, 100mg/d, oral, BID, combined with placebo gel, 9g/d, TID	78	61	27%	NR	NR	NR	EC	NR	NR	NR
		Placebo gel and tablet	79	61	30%							
Birbara 2006	NR (Study 1)	Celecoxib, 200mg/d, oral, OD	157	61	26%	NR	NR	NR	NR	NR	Yes	Yes
		Placebo	78	61	28%							
	NR (Study 2)	Celecoxib, 200mg/d, oral, OD	169	61	35%	NR	NR	NR	NR	NR	Yes	Yes
		Placebo	85	60	29%							
Case 2003	NR	Diclofenac sodium, 150 mg/d, oral, BID	25	63	40%	NR	NR	NR	EC	NR	No	No
		Acetaminophen, 4000 mg/d, oral, QID	29	62	48%							
		Placebo	28	62	61%							
Schnitzer 2005c-1	NR	Diclofenac, 150 mg/d, oral, BID	94	60	32%	6	NR	NR	EC	No	Yes	Yes
		Placebo	97	62	33%	8						
Schnitzer 2005c-2	NR	Diclofenac, 150 mg/d, oral, BID	91	54	23%	9	NR	NR	EC	No	Yes	Yes
		Placebo	99	55	31%	9						
Kivitz 2002		Naproxen, 1000 mg/d, oral, BID	205	60	37%	9	NR	NR	15%	No	Yes	NR
		Placebo	205	60	36%	8			10%			
Geusens 2004	NR	Naproxen, 1000mg/d, oral, BID	279	54	21%	11	NR	NR	EC	No	Yes	Yes
		Placebo	284	53	21%	9						

Saag 2000	NR	Ibuprofen, 2400mg/d, oral, TID	221	61	26%	10	NR	NR	EC	No	No	Yes
		Placebo	69	62	19%	9						

22854
