# Supplementary material for: High correlation of VAS pain scores after 2 and 6 weeks of treatment with VAS pain scores at 12 weeks in randomised controlled trials in rheumatoid arthritis and osteoarthritis: meta-analysis and implications
Source: Arthritis Res Ther. 2016 Mar 31;18:73. doi: 10.1186/s13075-016-0972-7 (PMC4818534; doi:10.1186/s13075-016-0972-7)
Supplement: Additional file 4: — Input data tables. (PDF 368 kb) [file 13075_2016_972_MOESM4_ESM.pdf]

Paper ID	Study ID	Arm ID	Author.Year	Treatments	N_ALL	CFB_2_m	CFB_2_se	N_6	CFB_6_m	CFB_6_se	N_12	CFB_12_m	CFB_12_se
1	1	1	Gibofsky 2003	Placebo, oral, OD, 6 weeks	98	-15.00	2.77	91	-21.20	2.88	NA	NA	NA
		2		Celecoxib, 200mg/d, oral, OD, 6 weeks	189	-29.00	1.98	178	-34.00	2.06	NA	NA	NA
2	2	3	Reginster 2007	Part I: Naproxen, 1000mg/d, 500mg twice daily	439	-26.90	2.00	NA	-30.40	1.90	439	-28.57	1.07
		4		Part I: Etoricoxib, 60mg/d, OD	446	-27.00	2.10	NA	-30.40	2.00	446	-27.94	1.07
3	3	5	Bingham 2007	Placebo	127	-7.70	2.05	NA	-13.30	2.30	126	-15.70	2.30
		6		Celecoxib, 200mg/d, oral, OD	241	-21.70	1.60	NA	-24.70	1.50	236	-25.00	1.80
		7		Etoricoxib, 30mg/d, oral, OD	231	-25.10	1.60	NA	-27.20	1.90	228	-28.90	1.70
3	4	8	Bingham 2007	Placebo	117	-11.30	2.30	NA	-16.30	2.40	112	-16.40	2.60
		9		Celecoxib, 200mg/d, oral, OD	247	-23.50	1.60	NA	-27.10	1.70	246	-28.00	1.80
		10		Etoricoxib, 30mg/d, oral, OD	244	-23.90	1.50	NA	-27.70	1.60	243	-27.70	1.80
4	5	11	Baerwald 2010	Placebo	331	-11.60	1.37	NA	-15.80	1.60	331	-17.97	1.68
		12		Naproxen, 1000mg/d, oral, BID	156	-23.90	1.79	NA	-26.70	2.10	156	-24.31	2.23
5	6	13	Day 2000	Placebo	74	-17.90	2.04	74	-18.92	2.40	NA	NA	NA
		14		Ibuprofen, 2400mg, NA, 800mg 3x per day	244	-32.90	1.02	249	-33.55	1.40	NA	NA	NA
6	7	15	Schnitzer 2005a	Placebo	104	-21.80	2.59	104	-26.00	2.69	NA	NA	NA
		16		Naproxen, 1000mg/d, oral,	117	-34.80	2.44	117	-37.90	2.54	NA	NA	NA

				500mg twice daily									
7	8	17	Sowers 2005	Celecoxib 200mg/d, once daily	136	NA	NA	136	-13.20	1.90	136	-15.20	2.00
		18		Naproxen 1000mg/d, 500mg twice daily	128	NA	NA	128	-12.80	2.00	128	-15.10	2.10
8	9	19	Schnitzer 2004	Placebo	97	-12.40	2.69	96	-16.80	2.80	NA	NA	NA
		20		Diclofenac, 150mg/d, oral, 75mg twice daily	94	-31.70	2.74	93	-33.40	2.85	NA	NA	NA
9	10	21	Tannenbaum 2004	Placebo	243	-9.10	1.24	243	-18.50	1.76	243	-19.80	1.67
		22		Celecoxib, 200mg/d, 200mg once daily	481	-17.50	0.93	481	-25.00	1.25	481	-25.20	1.13
10	11	23	Schnitzer 2010	Placebo b.i.d.	221	-19.60	1.66	NA	-23.00	1.70	221	-24.08	1.84
		24		Naproxen, 1000mg/d, 500mg, b.i.d.	227	-32.30	1.45	NA	-35.80	1.80	226	-36.51	1.81
11	12	25	Dahlberg 2009	Celecoxib, 200mg, oral, OD	458	NA	NA	458	-12.00	1.28	458	-11.00	1.19
		26		Diclofenac, 100mg, oral, BID	458	NA	NA	458	-13.00	1.28	458	-10.00	1.19
12	13	27	Schnitzer 2011b	Placebo (pooled arms)	257	-15.20	1.65	256	-19.00	1.72	256	-20.40	1.62
		28		Naproxen (1000 mg/d), 500 mg bid	254	-27.60	1.66	254	-30.90	1.72	254	-29.50	1.62
13	14	29	Smugar 2006	Placebo	150	-23.10	2.00	146	-17.60	1.90	NA	NA	NA
		30		Celecoxib, 200mg/d, oral, OD	456	-34.20	1.20	447	-33.00	1.00	NA	NA	NA
13	15	31	Smugar 2006	Placebo	151	-18.30	2.00	150	-16.70	1.80	NA	NA	NA
		32		Celecoxib, 200mg/d, oral, OD	460	-32.20	1.40	459	-30.80	1.00	NA	NA	NA
14	16	33	Schnitzer 2011a	Placebo	416	-20.60	1.29	416	-23.30	1.35	416	-24.40	1.24
		34		Celecoxib, 200mg/d, oral, 200mg once	419	-28.70	1.29	419	-32.60	1.34	419	-33.60	1.24

				daily									
15	17	35	Emery 2008	Celecoxib, 200mg/d, oral, OD	69	NA	NA	69	-20.00	2.80	50	-17.00	3.32
		36		Diclofenac, 150mg/d, oral, TID	72	NA	NA	72	-35.00	3.20	48	-30.00	3.80
16	18	37	Gibofsky 2007	Placebo	171	NA	NA	169	-16.00	2.11	169	-14.90	2.24
		38		Naproxen 1000/d, oral, 500mg 2x daily	167	NA	NA	166	-30.20	2.10	166	-30.80	2.22
17	19	39	McKenna 2001a	Placebo	200	-20.40	1.80	200	-23.10	2.00	NA	NA	NA
		40		Celecoxib, 200mg/d, 100mg bid	201	-33.40	1.91	199	-34.90	2.00	NA	NA	NA
		41		Diclofenac 150mg/d, 50mg TID	199	-35.50	1.94	199	-36.80	2.00	NA	NA	NA
18	20	42	Williams 2000	Placebo	232	-12.30	1.74	231	-14.00	1.81	NA	NA	NA
		43		Celecoxib, 200mg, oral, BID	231	-24.80	1.74	231	-27.30	1.81	NA	NA	NA
		44		Celecoxib, 200mg, oral, once daily	223	-25.90	1.77	222	-27.30	1.84	NA	NA	NA
19	21	45	Bocanegra 1998	Placebo	91	-11.60	2.67	91	-13.00	3.19	NA	NA	NA
		46		Diclofenac sodium, 150mg/d, oral, BID	154	-26.70	2.28	154	-28.70	2.48	NA	NA	NA
20	22	47	Wiesenhutter 2005	Placebo	104	-8.85	2.25	101	-18.20	2.20	101	-19.00	2.70
		48		Etoricoxib, 30mg/d, oral, 30mg once daily	214	-22.20	1.40	212	-27.90	1.60	212	-29.80	1.90
		49		Ibuprofen, 2400mg/d, oral, 800mg 3 times daily	210	-22.00	1.60	209	-25.90	1.50	209	-27.00	2.10
21	23	50	Schnitzer 2005b	Celecoxib, 200 mg, once daily	523	-27.60	1.10	514	-29.60	2.00	NA	NA	NA
		51		Acetaminophen 4000 mg, (1000mg QID)	269	-24.10	1.40	264	-24.60	1.80	NA	NA	NA
22	24	52	Sheldon 2005	Placebo	382	-13.90	1.35	382	-18.80	1.41	382	-18.10	1.31
		53		Celecoxib 200 mg/d	393	-24.30	1.33	393	-26.60	1.39	393	-24.10	1.33

23	25	54	Biegert 2004	Placebo	41	-6.00	4.12	41	-5.00	3.60	NA	NA	NA
		55		Diclofenac, 100mg/d, oral, BID, enteric coated	43	-23.00	4.02	43	-23.00	3.00	NA	NA	NA
24	26	56	Zhao 1999/Bensen 1999	Placebo	203	-13.50	1.85	203	-18.60	1.93	203	-17.00	1.78
		57		Celecoxib 100mg, 50mg b.i.d.	203	-17.50	1.85	203	-17.60	1.93	203	-15.60	1.78
		58		Celecoxib 200mg, 100mg b.i.d.	197	-29.00	1.88	197	-29.30	1.96	197	-27.00	1.81
		59		Celecoxib 400mg, 200mg b.i.d.	202	-26.60	1.86	202	-26.80	1.93	202	-24.80	1.78
		60		Naproxen 1000mg, 500mg b.i.d.	198	-28.90	1.87	198	-29.10	1.95	198	-25.00	1.80
25	27	61	DeLemos 2011	Placebo	202	NA	NA	200	-23.76	1.94	200	-18.98	1.78
		62		Celecoxib 200 mg/d, oral, OD	203	NA	NA	202	-32.18	1.93	202	-26.00	1.80
26	28	63	Fleischmann 2006	Placebo OD	231	-15.90	1.60	231	-19.70	1.81	231	-21.30	1.73
		64		Celecoxib 200 mg/d, OD	444	-20.30	1.09	444	-25.50	1.30	444	-27.40	1.32
27	29	65	Davies 1999	Placebo	50	-34.40	3.85	46	-5.70	4.05	NA	NA	NA
		66		Ibuprofen, 2400mg/d, oral, TID	54	-33.80	3.85	49	-16.20	3.92	NA	NA	NA
28	30	67	Lehmann 2005	Placebo	424	-11.30	0.91	424	-20.40	1.33	424	-21.40	1.16
		68		Celecoxib 200 mg, oral, 200mg od	420	-17.50	0.98	419	-26.70	1.34	420	-26.60	1.15
29	31	69	Altman 1998	Placebo	115	-20.00	2.09	149	-30.00	2.25	129	NA	NA
		70		Naproxen, 1000mg/d, oral, BID	113	-21.00	2.13	143	-29.00	2.30	125	NA	NA
30	32	71	Puopolo 2007	Placebo	111	-12.20	2.20	NA	-17.80	2.60	109	-16.47	2.08
		72		Etoricoxib, 30mg/d, 30mg qd	224	-22.20	1.70	NA	-30.00	2.10	220	-28.14	1.58
		73		Ibuprofen, 2400mg/d,Q99	213	-21.60	1.70	NA	-24.40	2.00	211	-24.10	1.58

				800mg, tid									
31	33	74	McKenna 2001b	Placebo	60	-23.80	3.41	60	-25.00	3.60	NA	NA	NA
		75		Celecoxib, 200mg/d, oral, once daily	63	-32.90	3.32	63	-39.00	3.60	NA	NA	NA
32	34	76	Hochberg 2011/Cryer 2011	Placebo	124	NA	NA	124	-32.10	2.47	108	-35.60	2.64
		77		Celecoxib 200 mg/d, oral, QD	242	NA	NA	242	-40.50	1.77	221	-41.80	1.85
32	35	78	Hochberg 2011/Cryer 2011	Placebo	122	NA	NA	122	-35.20	2.49	106	-38.40	2.67
		79		Celecoxib 200 mg/d, oral, QD	244	NA	NA	244	-39.20	1.76	220	-42.90	1.85
33	36	80	Williams 2001	Placebo	244	-12.40	1.69	243	-15.00	1.76	NA	NA	NA
		81		Celecoxib, 200mg/d, 100mg b.i.d.	243	-22.50	1.70	241	-21.20	1.77	NA	NA	NA
		82		Celecoxib, 200mg/d, 200mg q.d.	231	-21.10	1.74	231	-23.50	1.81	NA	NA	NA
34	37	83	Leung 2002	Placebo	56	-13.20	2.90	NA	-16.90	3.10	56	-15.33	2.74
		84		Naproxen 1000mg/d, 500mg twice daily	221	-25.90	1.60	NA	-25.10	1.90	221	-25.32	1.44
		85		Etoricoxib 60mg once daily	224	-24.50	1.50	NA	-25.70	1.80	224	-25.76	1.44
35	38	86	Makarowsky 2002	Placebo	118	-14.40	2.44	117	-16.00	2.54	117	-15.20	2.34
		87		Naproxen (1000mg/d), 500mg b.i.d	118	-27.30	2.43	118	-26.10	2.53	118	-22.00	2.33
36	39	88	Kivitz 2001	Placebo	218	-11.80	1.79	217	-13.20	1.86	217	-11.10	1.72
		89		Celecoxib 100 mg/day (in divided doses, twice daily)	216	-19.70	1.79	216	-21.50	1.87	216	-19.00	1.73
		90		Celecoxib 200 mg/day (in divided doses, twice daily)	207	-24.40	1.83	207	-25.10	1.91	207	-23.30	1.76
		91		Celecoxib 400 mg/day (in divided	213	-24.40	1.81	213	-23.90	1.88	213	-19.30	1.74

				doses, twice daily)									
		92		Naproxen 1000 mg/day (in divided doses, twice daily)	207	-26.50	1.83	207	-24.80	1.91	207	-22.30	1.76
37	40	93	Boswell 2008	Placebo	107	-11.90	2.00	101	-16.90	1.90	NA	NA	NA
		94		Celecoxib, 200mg/d, oral, OD	109	-14.10	1.90	105	-19.60	2.00	NA	NA	NA
37	41	95	Boswell 2008	Placebo	186	-14.80	1.50	NA	-24.40	2.10	167	-21.70	1.70
		96		Celecoxib, 200mg/d, oral, OD	185	-23.10	1.50	NA	-28.10	1.90	163	-28.00	1.80
38	42	97	Sandelin 1997	Placebo gel and tablet	79	-13.00	2.97	79	-21.00	3.09	NA	NA	NA
		98		Diclofenac, 100mg/d, oral, BID, combined with placebo gel, 9g/d, TID	78	-17.00	2.99	78	-22.00	3.11	NA	NA	NA
39	43	99	Birbara2006	Placebo	78	-17.60	5.60	78	-19.24	5.90	NA	NA	NA
		100		Celecoxib 200 mg/d, oral, 200mg once daily	157	-25.20	4.20	156	-27.30	4.50	NA	NA	NA
39	44	101	Birbara2006	Placebo	85	-18.90	5.80	81	-20.95	6.10	NA	NA	NA
		102		Celecoxib 200 mg/d, oral, 200mg once daily	169	-24.70	4.50	164	-28.51	4.70	NA	NA	NA
40	45	103	Case 2003	Placebo	28	-0.30	0.74	NA	NA	NA	28	-3.06	3.73
		104		Acetaminophen, (4000 mg/d), oral, QID	29	-0.94	0.80	NA	NA	NA	29	-4.76	3.09
		105		Diclofenac Sodium, (150 mg/d), oral, BID	25	-12.04	0.97	NA	NA	NA	25	-10.78	3.17
41	46	106	Schnitzer 2005-1	Placebo	96	-14.10	2.69	96	-19.00	2.80	NA	NA	NA
		107		Diclofenac, 150 mg/d, oral, BID	93	-30.50	2.74	93	-33.10	2.85	NA	NA	NA

42	47	108	Schnitzer 2005-2	Placebo	99	-16.00	2.66	98	-18.60	2.77	NA	NA	NA
		109		Diclofenac, 150 mg/d, oral, BID	91	-25.20	2.77	91	-24.60	2.88	NA	NA	NA
43	48	110	Kivitz 2002	Placebo	205	-21.19	1.84	205	-23.92	1.94	205	-25.97	2.07
		111		Naproxen, 1000 mg/d, oral, BID	205	-31.03	1.85	204	-31.84	1.93	204	-31.83	2.08
44	49	112	Geusens 2004	Placebo	284	-16.40	1.68	284	NA	NA	284	-19.30	1.38
		113		Naproxen, 1000mg/d, oral, BID	279	-22.60	1.62	279	NA	NA	279	-23.40	1.42
45	50	114	Saag 2000	Placebo	69	-11.70	2.87	69	-15.10	2.90	NA	NA	NA
		115		Ibuprofen, 2400mg/d, oral, TID	221	-24.80	1.60	221	-27.40	1.62	NA	NA	NA
